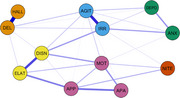# Network analysis of neuropsychiatric symptoms in behavioral variant frontotemporal dementia

**DOI:** 10.1002/alz70857_107382

**Published:** 2025-12-26

**Authors:** Grace J Goodwin, Sebastian Mehrzad, Samantha E John

**Affiliations:** ^1^ University of Nevada, Las Vegas, Las Vegas, NV, USA; ^2^ Princeton University, Princeton, NJ, USA; ^3^ University of Nevada Las Vegas, Las Vegas, NV, USA

## Abstract

**Background:**

Behavioral variant frontotemporal dementia (bvFTD) is characterized by neuropsychiatric symptoms (NPS) and personality changes that lead to functional decline and caregiver distress. There are currently no FDA‐approved pharmacotherapies for NPS in bvFTD, and existing medications, typically repurposed psychiatric and Alzheimer's agents, have limited efficacy in bvFTD. A more nuanced understanding of NPS in bvFTD is needed to inform individualized intervention. The present study uses network analysis to model NPS in bvFTD at initial clinic visit.

**Method:**

BvFTD patients were selected from the National Alzheimer's Coordinating Center (NACC) Uniform Data Set (UDS). Patients with other FTDs or related syndromes (e.g., corticobasal degeneration, progressive supranuclear palsy, primary progressive aphasia, amyotrophic lateral sclerosis, Alzheimer's disease) were excluded. The final sample (*n* = 1066) consisted of predominantly non‐Hispanic white (NHW=88.6%, HW=3.2%, NH Black=2.1%, HB = .2%, other=6%,), well‐educated (*M*_ed_=15.2[3.14]) older adults (*M*_age_=62.7[9.66]; 38.3% female). The NPS network consisted of 12 Neuropsychiatric Inventory Questionnaire (NPI‐Q) items: delusions, hallucinations, agitation/aggression, depression/dysphoria, anxiety, elation/euphoria, apathy/indifference, disinhibition, irritability/lability, motor disturbance, nighttime behaviors, appetite/eating problems. The *eLasso* method was used for network estimation, where “nodes” represent binary NPI‐Q items (e.g., symptom absent=0, symptom present=1) and “edges” represent their pairwise dependency. Node strength was calculated to determine relative importance of each NPI‐Q item to overall network connectivity.

**Result:**

Most of the sample met criteria for dementia (94.5%; mild cognitive impairment=5.5%). The most frequently endorsed symptom was apathy/indifference (79.01% endorsed) followed by disinhibition (67.70%). The NPI‐Q network (*M* = .197) consisted of positive edges, the strongest of which connected items within similar symptom domains. Four symptom clusters were identified: 1) hallucinations and delusions; 2) agitation/aggression and irritability/lability; 3) elation and disinhibition; 4) depression/dysphoria and anxiety; 5) apathy/indifference, appetite/eating problems, motor disturbance; 6) nighttime behaviors. Irritability/lability and agitation/aggression appeared to have highest node strength; however, centrality values were not stable and should be interpreted with caution.

**Conclusion:**

NPS in bvFTD are highly interconnected—endorsing one NPS increases the likelihood of endorsing other NPS. Interventions targeting irritability and agitation may decrease symptom connectivity and “contagion”. Larger samples are needed to ensure stable centrality values and confirm network features. Findings may inform clinical presentations and management of NPS in bvFTD.